# Blood-Based DNA Methylation Biomarkers for Type 2 Diabetes: Potential for Clinical Applications

**DOI:** 10.3389/fendo.2018.00744

**Published:** 2018-12-04

**Authors:** Tarryn Willmer, Rabia Johnson, Johan Louw, Carmen Pheiffer

**Affiliations:** ^1^Biomedical Research and Innovation Platform, South African Medical Research Council, Tygerberg, South Africa; ^2^Division of Medical Physiology, Faculty of Health Sciences, Stellenbosch University, Tygerberg, South Africa; ^3^Department of Biochemistry and Microbiology, University of Zululand, Kwa-Dlangezwa, South Africa

**Keywords:** global DNA methylation, gene-specific DNA methylation, genome-wide DNA methylation, blood, type 2 diabetes, biomarkers

## Abstract

Type 2 diabetes (T2D) is a leading cause of death and disability worldwide. It is a chronic metabolic disorder that develops due to an interplay of genetic, lifestyle, and environmental factors. The biological onset of the disease occurs long before clinical symptoms develop, thus the search for early diagnostic and prognostic biomarkers, which could facilitate intervention strategies to prevent or delay disease progression, has increased considerably in recent years. Epigenetic modifications represent important links between genetic, environmental and lifestyle cues and increasing evidence implicate altered epigenetic marks such as DNA methylation, the most characterized and widely studied epigenetic mechanism, in the pathogenesis of T2D. This review provides an update of the current status of DNA methylation as a biomarker for T2D. Four databases, Scopus, Pubmed, Cochrane Central, and Google Scholar were searched for studies investigating DNA methylation in blood. Thirty-seven studies were identified, and are summarized with respect to population characteristics, biological source, and method of DNA methylation quantification (global, candidate gene or genome-wide). We highlight that differential methylation of the *TCF7L2, KCNQ1, ABCG1, TXNIP, PHOSPHO1, SREBF1, SLC30A8*, and *FTO* genes in blood are reproducibly associated with T2D in different population groups. These genes should be prioritized and replicated in longitudinal studies across more populations in future studies. Finally, we discuss the limitations faced by DNA methylation studies, which include including interpatient variability, cellular heterogeneity, and lack of accounting for study confounders. These limitations and challenges must be overcome before the implementation of blood-based DNA methylation biomarkers into a clinical setting. We emphasize the need for longitudinal prospective studies to support the robustness of the current findings of this review.

## Introduction

Diabetes mellitus is a leading cause of death and disability worldwide, affecting 415 million people in 2017, and this figure is expected to increase to 592 million by 2035 (1, 2). Type 2 diabetes (T2D) accounts for over 90% of diabetes mellitus cases and its incidence is increasing globally in response to escalating rates of obesity and insulin resistance. Indeed, according to the World Health Organization, over 90% of patients with T2D are overweight or obese (3). T2D is a progressive, chronic disorder with a long asymptomatic phase. The early stages of disease can remain undetected for many years, during which time micro- and macro-vascular complications may occur (4, 5). Identification of individuals during the asymptomatic phase would not only permit opportunities for early interventions to prevent the development of overt diabetes, but may also lead to better management of the disease. A major area of current research has thus been to search for robust, sensitive, and readily accessible biomarkers of T2D. In this regard, a striking amount of evidence has accumulated to suggest that changes to the epigenetic landscape in insulin-responsive tissues play an important role in the pathogenesis of obesity, insulin resistance, and T2D, and if reflected in blood, may represent potential biomarker candidates (6–8).

Epigenetics is defined as heritable changes that affect gene expression without altering the underlying genomic sequence (9). These processes include DNA methylation, chromatin modifications such as histone acetylation and methylation, and non-coding RNAs that act as regulatory molecules (9). DNA methylation is the most widely studied and best characterized epigenetic mechanism, and involves the covalent addition of a methyl group to carbon C5 of cytosine nucleotides to create 5-methylcytosine (5 mC) (10). Cytosine methylation occurs in cytosine-guanine dinucleotides (CpG) sites, which tend to cluster together as repetitive sequences known as CpG islands, which are primarily found within promoter regions of genes, or regions with increased centromeric tandem repeat units (9, 10). CpG methylation within promoter regions is generally associated with gene silencing, although recent studies have provided evidence of the importance of non-CpG and non-promoter methylation in development and disease (11, 12). DNA methylation alterations can occur in response to biological (13, 14), lifestyle (15, 16), and environmental (17) factors and associate with gene expression changes and pathological dysfunctions. Moreover, DNA methylation is reversible, and therefore aberrant DNA methylation modifications have attracted increased interest as drug targets. As such, the interest in dissecting the impact of epigenetic variation on human diseases, including T2D, has increased over the last decade (18–23).

For numerous epidemiological studies, it is not always possible to access human tissues central to the pathogenesis of disease. Importantly, T2D-associated DNA methylation changes in pancreatic ß-cells and insulin-responsive tissues (liver, muscle, and adipose tissue) have been reported to be reflected in the blood, thus offering an opportunity to use alternative, non-invasive clinical samples for methylation analysis (7, 8, 24). Peripheral blood is relatively quick and easy to collect, with minimal side effects and much higher patient acceptability. As blood collection is already a part of routine medical checkups in both developed and developing countries, identification of blood-based DNA methylation alterations would greatly facilitate biomarker discovery for T2D screening. This, together with the chemical and biological stability of DNA methylation signatures, mark them as attractive and feasible prognostic/diagnostic tools.

Despite the exponential rise in epigenetic research over the last decade, the current status of DNA methylation alterations in blood from human T2D subjects still remains limited. Indeed, most recent reviews have focused on DNA methylation signatures in pancreatic islets and peripheral tissues (liver, skeletal muscle, and adipose tissue), which are not feasible tissue sources for biomarker generation. The aim of this review is to summarize, discuss, and integrate the most recent available evidence of the potential of blood-based DNA methylation alterations as candidate biomarkers for T2D prevention and treatment.

Four databases, Scopus, PubMed, Cochrane Central, and Google Scholar, were searched to identify published studies reporting DNA methylation changes in blood between January 2008 to July 2018. The following keywords: “DNA methylation,” AND “blood,” OR “peripheral blood,” OR “peripheral blood mononuclear cells,” OR “peripheral blood leukocytes” OR “peripheral blood lymphocytes” OR “white blood cells” AND “type 2 diabetes” AND “human” were used. Studies were considered eligible if they were original articles, investigated DNA methylation patterns in relationship with T2D and if the study was published in English. Reference lists of included studies were also hand-searched to identify other potentially eligible studies. Both cross-sectional, case control, and longitudinal studies that provided sufficient information were included. The study methods included global DNA methylation studies, candidate-gene methylation studies, and genome-wide association studies (GWAS).

## Global DNA Methylation Studies

Global DNA methylation, referred to as the total methylation status that occurs across the genome, has been reported to be one of the earliest molecular changes in the transition of a cell from a normal to a diseased state (25). Technological advances have resulted in an increase in global DNA methylation studies. Current methods to quantify global DNA methylation include enzyme-linked immunosorbent assays (ELISA), methylation-sensitive restriction enzymes, liquid chromatography coupled with mass spectrometry, flow cytometry, and quantification of DNA methylation within repetitive elements using bisulfite pyrosequencing (26). Global DNA methylation studies are advanced in cancer research, with a blood-based candidate diagnostic biomarker for colorectal cancer already commercially available (27). In recent years, global DNA has attracted considerable interest as a biomarker for T2D. Studies that have quantified global DNA methylation in peripheral blood of T2D subjects are summarized in Table 1.

**Table 1:** Main findings from T2D studies investigating global DNA methylation in blood.

**Author (year)**	**Country**	**Sample size**	**Gender**	**Biological source**	**Method**	**Study outcome**
Luttmer et al. (28)	Netherlands	IGT = 172 T2D = 286 Controls = 280	M and F	PBL	5 mC/C ratio measurement by LCMS	Global DNA hypomethylation in IGT and individuals with T2D compared to control subjects. Methylation negatively associated with fasting blood glucose concentrations and positively associated with HDL.
Pinzon-Cortes et al. (29)	Colombia	T2D = 44 Controls = 35	Unknown	PB	5 mC measurement using colorimetric methylated DNA quantification	Global hypermethylation in patients with T2D compared to controls.
Matsha et al. (30)	South Africa	IGT = 119 T2D = 158 Controls = 287	M and F	PBMCs	5 mC measurement using Imprint DNA methylation ELISA	Global hypermethylation in pre-diabetic and treatment naïve T2D individuals compared to controls while no significant difference observed in global DNA methylation between individuals with T2D on treatment and those with normoglycaemia. NOS3 G894T polymorphism an independent determinant of global DNA methylation.
Simar et al. (31)	Denmark	T2D = 12 Obese = 14 Controls = 7/11	M	PBMCs/monocytes, lymphocytes/T cells	5 mC measurement using bead-based flow cytometry	Increased global DNA methylation levels in B cells from obese and T2D subjects and in natural killer lymphocytes from T2D patients. No overall association between PBMC methylation levels and T2D/obesity.
Zhang et al. (32)	China	T2D = 75 Controls = 29	M and F	PB	5 mC measurement using HPLC	No association between DNA methylation and T2D between groups.
Martin-Nunez et al. (33)	Spain	T2D = 12 Controls = 12	M	PB	LINE-1 measurement using pyrosequencing	LINE-1 DNA methylation inversely correlated with T2D risk.
Pearce et al. (34)	England	228 non-diabetic	M and F	PB	LINE-1 measurement using pyrosequencing	Increased methylation associated with increasing fasting glucose concentrations, total cholesterol, total triglycerides, and LDL cholesterol. No differences in LINE-1 methylation between M and F.
Wu et al. (35)	China	T2D = 205 Controls = 213	M and F	PBL	Quantitative methylation-specific PCR	LINE-1 DNA methylation positively correlated with T2D risk.
Zhao et al. (36)	Vietnam	84 monozygotic twin pairs, 11.4% diabetic	M	PBL	*Alu* repetitive elements using pyrosequencing	Global *Alu* hypermethylation positively associated with insulin resistance.
Thongsroy et al. (37)	Thailand	IGT = 113 T2D = 85 Controls = 42	M and F	WBC	*Alu* repetitive elements using COBRA	Global *Alu* hypomethylation positively associated with high fasting blood glucose, HbA1c and high blood pressure.

*Alu, Arthrobacter luteus; COBRA, ALU-Combined Bisulfite Restriction Analysis; ELISA, enzyme-linked immunosorbent assay; HbA1c, glycated hemoglobin A1c; HDL, High-density lipoprotein; HPLC, high-performance liquid chromatography; IGT, impaired glucose tolerance; LCMS, Liquid chromatography mass spectrometry; LDL, Low-density lipoprotein; LINE-1, Long interspersed nuclear element-1; NOS3, nitric oxide synthase 3; PB, Peripheral blood; PBL, Peripheral blood leukocytes; PBMCs, Peripheral blood mononuclear cells; T2D, Type 2 Diabetes; WBC, White blood cells; 5 mC, 5 methyl cytosine*.

Luttmer et al. quantified global DNA methylation levels in peripheral blood leukocytes of 738 individuals from the Netherlands Hoorn Study cohort and reported a progressive decrease in global DNA methylation in individuals with T2D compared to those with impaired glucose tolerance and normoglycaemia. Moreover, DNA hypomethylation in these subjects was independently associated with hyperglycaemia and high-density lipoprotein (HDL) cholesterol (28). In contrast, a Colombian study using a smaller patient group, observed a global increase in DNA methylation in 44 subjects with T2D compared to 35 healthy controls, which correlated with the percentage of glycated hemoglobin A1c (HbA1c) (29). Similar findings were reported by Matsha et al. using a South African population consisting of 158 individuals with T2D, 119 with dysglycaemia, and 287 healthy controls. They showed that levels of global DNA methylation were higher in individuals with impaired glucose tolerance or treatment-naïve T2D compared to those with normoglycaemia (29, 30). Interestingly, no difference in global DNA methylation was observed between diabetic individuals on treatment and normoglycaemic subjects, prompting the authors to speculate that glucose management caused the reversal of aberrant DNA methylation patterns during T2D (30).

An innovative study by Simar et al. measured global DNA methylation levels of different subtypes of peripheral blood mononuclear cells in obese and T2D individuals, using a flow cytometry bead-based method. They reported that global DNA methylation levels were increased in B cells from obese individuals and subjects with T2D, and in natural killer lymphocytes from patients with T2D, while no overall difference was observed in the mixed population of blood mononuclear cells from these individuals. DNA methylation in B cells and natural killer lymphocytes correlated positively with insulin resistance, suggesting an association between DNA methylation alterations, immune function, and metabolic disorders (31). These findings highlight the importance for not only tissue specific but also cell type specific epigenetic studies, which may improve sensitivity and specificity.

Repetitive elements, such as *long interspersed nuclear element-1* (*LINE-1*) are highly represented throughout the genome and as such, methylation of these elements is generally considered to correlate with global genomic DNA methylation (38). Martin-Núñez (33) demonstrated that *LINE-1* methylation was decreased in peripheral blood from a small Spanish group of 12 individuals with T2D compared to 12 normoglycaemic individuals (33). Conversely, a study using 228 non-diabetic individuals reported that *LINE-1* DNA hypermethylation is associated with increasing fasting glucose, total cholesterol, triglycerides, low-density lipoprotein (LDL) cholesterol, and risk of developing T2D (34). These findings were later supported by Wu and colleagues who reported a significant increase in *LINE-1* methylation in a group of 205 Chinese patients with T2D compared to 213 healthy controls (35). Using a similar approach, Zhao and colleagues assessed methylation of *Arthrobacter luteus* (*Alu*) elements as a proxy for global DNA methylation by performing quantitative bisulfite pyrosequencing. They found that hypermethylation of these elements in peripheral blood leukocytes from 84 monozygotic twin pairs discordant for T2D was significantly associated with insulin resistance (36). More recently, *Alu* methylation levels were also investigated in white blood cells from 85 individuals with T2D, 113 with impaired glucose tolerance, and 42 healthy control subjects, using *Alu*-Combined Bisulfite Restriction Analysis (COBRA). Interestingly, in contrast to the findings of Zhao et al. (36), they reported that individuals with T2D exhibited the lowest *Alu* methylation compared to controls, which directly correlated with higher fasting blood glucose and HbA1c concentrations and high blood pressure (37). Taken together, the results from global DNA methylation analysis are mostly inconsistent and more studies are needed to consolidate the findings on the association between blood-based global DNA methylation and T2D.

## Candidate Gene Studies

Despite being a robust measure of overall genomic methylation, global DNA does not have the resolution to measure methylation within specific genes (39). Thus, a candidate gene approach to quantify the methylation status of specific CpG sites within genes associated with T2D are increasingly being investigated (40). Methods used in these studies include methylated DNA immunoprecipitation (MeDIP), methylation specific PCR, mass spectrometry combined with RNA base-specific cleavage, as well as bisulfite pyrosequencing (41). Genes investigated include *fat mass and obesity-associated protein* (*FTO*), *peroxisome proliferator–activated receptor gamma* (*PPAR*γ), *pyruvate dehydrogenase lipoamide kinase isozyme 4* (*PDK4*), *transcription factor 7-like 2* (*TCF7L2*), *monocyte chemoattractant protein-1* (*MCP-1*), *glucokinase* (*GCK*), *protein kinase C zeta* (*PRCKZ*), *B-cell lymphoma/leukemia 11A* (*BCL11A*), *gastric inhibitory polypeptide receptor* (*GIPR*), *solute carrier family 30 member 8* (*SLC30A8*), *insulin-like growth factor-binding protein 7* (*IGFNP-7*), *protein tyrosine phosphatase, non-receptor type 1* (*PTPN1*), *calmodulin 2* (*CALM2*), *CRY2 cryptochrome circadian regulator 2* (*CRY2*), *Ca2*+*/calmodulin-dependent protein kinase 1 subfamily of serine/threonine kinases* (*CAMK1D*), *toll-like receptor* (*TLR*) *2*, and *4*, and *free fatty acid receptor 3* (*FFAR3*), which are discussed in further detail below and summarized in Table 2

**Table 2:** Main findings from T2D studies investigating candidate gene methylation in blood.

**Author (year)**	**Genes investigated**	**Country**	**Sample size**	**Gender**	**Tissue type**	**Method**	**Study outcome**
van Otterdijk et al. (40)	*KCNJ11, PPARγ, PDK4, KCNQ1, SCD1, PDX1, FTO* and *PEG3*	Germany	T2D = 25 Controls = 11	M and F	PBL	Bisulphite pyrosequencing	Hypermethylation of *FTO*, hypermethylation of *PPARγ*, and hypomethylation *PDK4* associated with metabolic syndrome, T2D and both metabolic syndrome and T2D, respectively.
Canivell et al. (42)	*TCF7L2* (22 CpGs)	Spain	T2D = 93 Controls = 93	M and F	WB	LCMS and RNA base-specific cleavage	Hypermethylaion of 8 CpGs and hypomethylation of five CpGs were observed in T2D patients compared to controls. Differential methylation of CpGs at −382, +5, +96, and +186 (relative to ATG) associated with fasting glucose and CpG at +137 associated with total cholesterol and LDL-cholesterol.
Liu et al. (43)	*MCP-1*	China	T2D = 32 Controls = 15	M and F	PBMCs	Methylation specific PCR	Hypomethylation of *MCP-1* in T2D patients compared to controls.
Tang et al. (44)	*GCK* (4 CpGs)	China	T2D = 48 Controls = 48	M and F	PB	Bisulfite pyrosequencing	Hypermethylation of one CpG site in *GCK* in T2D subjects compared to controls. Association specific to males.
Zou et al. (45)	*PRKCZ* (9 CpGs)	China	T2D = 152 Controls = 120	M and F	PBL	Bisulfite pyrosequencing	Hypermethylation of seven CpG sites in T2D patients compared to controls.
Tang et al. (46)	*BCL11A* (5 CpGs)	China	T2D = 48 Controls = 48	M and F	PB	Bisulfite pyrosequencing	Significant association between mean DNA hypomethylation of *BCL11A* CpGs and T2D in males but not females.
Canivell et al. (47)	*GIPR*	Spain	T2D = 93 Controls = 93	M and F	WB	LCMS and RNA base-specific cleavage	Hypomethylation of *GIPR* promoter associated with increased fasting blood glucose levels and HOMA-IR.
Seman et al. (48)	*SLC30A8* (6 CpGs)	Malaysia	T2D = 509 Controls = 441	M and F	PB	Bisulfite pyrosequencing	Hypermethylation at five CpGs in T2D subjects compared to controls. Combined methylation scores of all 6 CpGs significantly increased in T2D subjects compared to controls.
Gu et al. (49)	*IGFBP-7* (3 CpGs)	Sweden	T2D TN = 100 T2D T = 140 Controls = 100	M and F	PB	Bisulfite-pyrosequencing	Hypermethylation of three CpG sites observed in newly diagnosed, treatment naïve T2D patients compared to controls. Combined methylation scores from all three CpGs showed increased genomic methylation levels in T2D compared to normoglycaemic controls.
Huang et al. (50)	*PTPN1* (8 CpGs)	China	T2D = 97 Controls = 97	M and F	PBMCs	Bisulfite-pyrosequencing	Hypermethylation of all eight CpGs correlated with T2D risk and inversely associated with low-density lipoprotein and total cholesterol in females.
Cheng et al. (51)	*CAMK1D* (9 CpGs), *CRY2* (5 CpGs), *CALM2* (4 CpGs)	China	T2D = 48 Controls = 48	M and F	PB	Bisulfite pyrosequencing	Hypomethylation in promoters of all three genes observed in T2D subjects compared to controls.
Remely et al. (52)	*TLR2* (7 CpGs), *TLR4* (4 CpGs)	Austria	T2D = 24 Obese = 14 Controls = 18	M and F	WB	Bisulfite pyrosequencing	Mean methylation of all four CpGs in the first exon of *TLR4* were significantly reduced in obese subjects compared to T2D subjects, while no differences in mean methylation were observed between T2D subjects and lean controls. Reduced methylation of seven CpGs in the *TLR2* promoter observed in T2D vs. lean group, while no differences observed between obese group and lean controls.
Remely et al. (52)	*FFAR3*	Austria	T2D = 24 Obese = 14 Controls = 18	M and F	WB	Bisulfite pyrosequencing	Significantly reduced methylation in T2D subjects compared to controls.

*BCL11A, B-cell lymphoma/leukemia 11A; CALM2, calmodulin 2; CAMK1D, Ca2+/calmodulin-dependent protein kinase 1 subfamily of serine/threonine kinases; CRY2, CRY2 cryptochrome circadian regulator 2, FFAR3, free fatty acid receptor 3; FTO, fat mass and obesity-associated protein; GCK, glucokinase; GIPR, gastric inhibitory polypeptide receptor; HOMA-IR, homeostatic model assessment-insulin resistance; IGFNP-7, insulin-like growth factor-binding protein 7; IGT, impaired glucose tolerance; LCMS, Liquid chromatography mass spectrometry; MCP-1, monocyte chemoattractant protein-1; PB, Peripheral blood; PBL, Peripheral blood leukocytes; PBMCs, Peripheral blood mononuclear cells; PDK4, pyruvate dehydrogenase lipoamide kinase isozyme 4; PPARγ, peroxisome proliferator–activated receptor gamma, PRCKZ, protein kinase C zeta; PTPN1, protein tyrosine phosphatase, non-receptor type 1; SLC30A8, solute carrier family 30 member 8; TCF7L2, transcription factor 7-like 2; TLR2, toll-like receptor 2, TLR4, toll-like receptor 4. T2D, Type 2 Diabetes; WB, Whole blood*.

### Genes Involved in Glucose and Lipid Metabolism

The *FTO* gene encodes a 2-oxoglutarate-dependent nucleic acid demethylase and various studies have reported that variants in the *FTO* locus are strongly linked with obesity and can predict risk of T2D and cardiovascular disease (53–57). The methylation status of *FTO* was analyzed by van Otterdijk et al. who identified hypermethylation of one CpG locus in the promoter region in peripheral blood leukocytes from 25 individuals with T2D compared to 11 control healthy subjects (40). The same study also identified hyper- and hypomethylation of CpG sites in the promoters of the *PPAR*γ and *PDK4* genes, respectively, in patients with T2D vs. healthy controls. PPARγ is a transcription factor that plays major roles in adipogenesis and insulin sensitivity, and agonists are currently being used as anti-diabetic agents (58). PDK4 is reported to play a role in the regulation of glucose metabolism and mitochondrial function, and hypomethylation of this gene has also been reported in skeletal muscle of diabetic patients compared to controls (59). TCF7L2 is involved in glucose homeostasis and was reported to be differentially methylated in 13 of its promoter CpGs (eight hypermethylated and five hypomethylated) between treatment-naïve patients with T2D and matched controls (42). Furthermore, methylation at specific CpG sites of the *TCF7L2* promoter correlated significantly with fasting glucose concentrations, total cholesterol, LDL-cholesterol, as well as the homeostatic model assessment for insulin resistance (HOMA-IR) (47).

MCP-1 is a chemokine that regulates macrophage migration and infiltration into adipose tissue and in this way, contributes to insulin resistance and decreased glucose uptake during obesity and T2D (60). Interestingly, hypomethylation of the *MCP-1* promoter associated with increased serum MCP-1 levels, HbA1c, and fasting blood glucose levels in patients with T2D compared to healthy controls (43). The *GCK* gene encodes glucokinase, a key glycolytic enzyme that catalyzes the first step in hepatic and pancreatic islet glucose utilization pathways (61). Tang et al. (44) evaluated *GCK* methylation in T2D subjects and matched controls, and identified significant hypermethylation of one intragenic CpG site exclusively in male patients with T2D compared to healthy controls, which also correlated with total cholesterol levels. The results for this association indicate an interaction between gender and T2D-associated methylation alterations.

### Genes Involved in Insulin Secretion and Function

PRKCZ, a member of the PKC family of serine/threonine kinases, functions downstream of phosphatidylinositol 3-kinase (PI3K) to positively regulate the insulin signaling pathway and contributes to the translocation of glucose transporter type 4 (GLUT4) from the cytoplasm to the membrane, where it facilitates glucose uptake (62). A comparison between the *PRKCZ* promoter sequence in peripheral blood leukocytes from Chinese individuals with either T2D or normoglycaemia showed that seven CpG sites were methylated in the T2D group whereas only one CpG site was methylated in the control group (45). Furthermore, the protein expression levels of PRKCZ in the serum of the group with T2D was significantly reduced compared to the control group, suggesting that *PRKCZ* promoter activity and gene expression are regulated by methylation (45). The *BCL11A* gene, encoding a CH2H2 type zinc-finger transcription factor, has also been associated with T2D risk. BCL11A plays a normal physiological role in lymphocyte production but variants of this gene have been shown to affect insulin response to glucose, as well glucagon secretion (63, 64). Tang et al. investigated the correlation between *BCL11A* methylation at one intragenic and four promoter CpG sites and T2D risk (46). They found a significant decrease in the mean DNA methylation levels across these CpG sites in males with T2D compared to normoglycaemic controls (46). Interestingly, these differences were not observed in females.

The *GIPR* gene encodes a receptor of the incretin, GIP, a gastrointestinal hormone that stimulates insulin response after ingesting food. Canivell et al. performed DNA methylation profiling of the *GIPR* promoter in peripheral blood DNA and identified differential methylation at nine CpG sites located upstream of the first exon between patients with T2D and controls. On average, these nine CpG sites were hypomethylated in patients with T2D and significantly correlated with waist circumference and fasting glucose concentrations (47). *SLC30A8* encodes a pancreas-specific, zinc efflux transporter, and reduced levels or activity of SLC30A8 hinder glucose-induced insulin secretion, as zinc is required for the crystallization of insulin within secretory granules (65). Seman et al. analyzed DNA methylation alterations in the *SLC30A8* promoter in peripheral blood from a large Malay population and identified hypermethylation of five CpG sites in patients with T2D compared to controls (48).

Another insulin associated gene that has been linked to T2D is *IGFBP-7*, a member of the insulin growth factor binding family. The expression of IGFBP-7 was previously found to be increased in the serum of subjects with T2D compared to controls, and significantly associated with insulin resistance (49, 66, 67). Gu et al. studied the correlation between *IGFBP-7* promoter methylation and T2D in peripheral blood from a large Swedish cohort of subjects. They identified increased *IGFBP-7* methylation in three CpG sites in newly diagnosed men with T2D, but not in women, compared to non-diabetic individuals (49). Interestingly, no significant differences in serum IGFBP-7 expression were observed between groups (49). These results are conflicting with previous studies that reported increased serum IGFBP-7 concentrations in T2D subjects compared to healthy individuals. The discrepancies in these results may be due to the use of a much larger sample size in the study by Gu et al. (140 T2D subjects and 100 controls), as well as differences in the selection criteria of the patients with T2D between studies (49, 66, 67). While Gu et al. (49) studied newly diagnosed, treatment naïve T2D subjects, the previous studies included participants on chronic pharmacological therapies, including insulin, oral hypoglycaemic agents, statins, fibrates, blood pressure–lowering agents, and aspirin (49, 66, 67).

More recently, Huang et al. investigated the methylation status of another key regulator of the insulin signaling pathway, *PTPN1*, in relation to T2D susceptibility (50). *PTPN1* encodes the protein-tyrosine phosphatase 1B protein, which attenuates the insulin signaling pathway by decreasing the phosphorylation of the insulin receptor and/or insulin receptor substrate 1 (68). In the study, DNA methylation of the *PTPN1* promoter region was quantified in peripheral blood mononuclear cells from 97 Chinese patients with T2D and 97 age- and gender-matched healthy controls, using bisulfite pyrosequencing. The results revealed a significant correlation between *PTPN1* promoter methylation and increased T2D risk in females, but not in males. Furthermore, *PTPN1* methylation was also inversely associated with low-density lipoprotein and total cholesterol levels in females. These results indicate that *PTPN1* promoter hypermethylation is a risk factor for T2D in the female Chinese population.

### Genes Associated With Pancreatic and Cardiovascular Function

Cheng et al. investigated DNA methylation in the promoters of *CALM2, CRY2*, and *CAMK1D*, based on previous reports linking variants of these genes with T2D susceptibility (51). They demonstrated that four, five, and nine CpGs within the *CALM2, CRY2*, and *CAMK1D* gene promoters, respectively, were significantly hypomethylated in the peripheral blood of subjects with T2D compared to healthy controls. CRY2 plays a role in circadian rhythm which, when desynchronized, results in metabolic disturbances including increased insulin and postprandial glucose levels, increased arterial blood pressure, and decreased leptin levels, which may predispose individuals to T2D (69). Variants in the *CALM2* gene, a member of the calmodulin family, have been associated with dialysis survival in T2D-associated renal disease, as well as arrhythmia susceptibility in infants (70). CAMK1D plays a key role in granulocyte function and reactive oxygen species (ROS) inhibition through the chemokine signal transduction pathway, and consequently, non-functional variants or hypomethylation of this gene may result in apoptosis and consequently, reduced β-cell mass (71).

### Genes Associated With Gut Microbiota

Remely et al. investigated DNA methylation of two genes involved in innate immunity and inflammation, *TLR 2*, and *4*, in response to changes in gut microbiota in individuals with T2D (72–74). This investigation was prompted by a spate of recent studies to show that changes in gut microbiota composition can lead to chronic low-grade inflammation, metabolic dysregulation, and T2D (75, 76). Remely et al. investigated three groups of subjects: patients with T2D using glucagon-like peptide-1 (GLP-1) agonist therapy, obese individuals without established insulin resistance, and a normal-weight control group. The authors identified four significantly hypomethylated CpGs in the first exon of *TLR4* in obese individuals compared to healthy controls, while methylation of seven CpGs in the promoter region of *TLR2* was significantly lower in subjects with T2D compared to obese subjects and normal-weight controls, which correlated with body mass index (BMI) (52). Furthermore, distinct changes in gut microbiota composition were observed between the three groups, the most significant being a high abundance of lactic acid bacteria in individuals with T2D.

Gut microbiota contribute to energy metabolism through the production of short chain fatty acids (SCFA) during fermentation in the colon. SCFAs are believed to alter DNA methylation patterns of genes involved in inflammatory reactions, including genes encoding free fatty acid receptors (FFARs) (77, 78). Based on this as well as their previous findings, a follow up study conducted by Remely et al. investigated the effect of gut microbiota and SCFA production on DNA methylation of *FFAR3* in blood from the same cohort described previously (52). Their results showed differential composition of gut microbiota in the T2D and obese subjects, and significantly higher methylation in five CpGs in the *FFAR3* promoter region in normal-weight controls compared to obese subjects with the lowest methylation in subjects with T2D (77). Taken together, these two studies provide evidence that differential composition of gut microbiota in obesity and T2D is associated with epigenetic gene regulation. The authors thus proposed that improvements in diet targeted to restore gut microbial balance may ameliorate aberrant epigenetics and be effective as a preventative treatment for metabolic syndrome (52).

## Genome-Wide Association Studies

With technological advances, the focus of epigenetics studies has shifted from candidate regions to high throughput, genome wide association studies (GWAS). In the past few years, as a result of a widespread use of techniques, including the Infinium Beadchip Arrays and methylation pull-down sequencing assays, major insights into DNA methylation changes associated with T2D have been obtained, which are summarized in Table 3.

**Table 3:** Main findings from T2D studies investigating genome-wide DNA methylation in human population-based studies.

**Author (year)**	**Population**	**Sample size**	**Gender**	**Tissue type**	**Method**	**Study outcome**
Toperoff et al. (79)	Jewish	T2D = 710 Controls = 459	M and F	WB	Microarray-based methylation assays	Differential methylation identified in 13 CpGs, mapping to *SLC30A8, TCF7L2, KCNQ1, FTO, THADA*, and *JAZF1* genes in T2D subjects compared to controls.
Chambers et al. (80)	Indian Asian and European	Indian Asian: T2D = 1,608 Controls = 11 927 European: T2D = 306 Controls = 6,760	M and F	PB	450 K	Differential methylation identified in five regions mapping to *TCF7L2, FTO, KCNQ1, TXNIP ABCG1, PHOSPHO1, SOCS3*, and *SREBF1* genes, replicated in two cohorts.
Dayeh et al. (81)	European	T2D = 19 Controls = 19	M and F	WB	450 K	*ABCG1, PHOSPHO1* associated with future T2D risk but not *SOCS3, SREBF1* or *TXNIP*. *ABCG1* hypermethylation positively associated with HbA1c and fasting insulin levels.
Kriebel et al. (82)	German	1,448 non-diabetic (FBG and HbA1c) 1,440 non-diabetic (FI and HOMA-IR) 617 non-diabetic (2-h insulin)	M and F	WB	450 K	DNA methylation at cg06500161 (*ABCG1*) associated with fasting glucose, fasting insulin, and HOMA-IR.
Hidalgo et al. (83)	American	^a^Healthy individuals = 544 ^b^Healthy individuals = 293	M and F	WB	450 K	*ABCG1* hypermethylation associated with fasting insulin and HOMA-IR.
Walaszczyk et al. (84)	Dutch	T2D = 100 Controls = 100	M and F	WB	450 K	Differential methylation of *ABCG1, LOXL2, TXNIP, SLC1A5*, and *SREBF1* associated with T2D.
Muftah et al. (19)	^a^Arab, ^b^Caucasian	^a^T2D = 30 Controls = 93 ^b^180 twins from TwinsUK cohort	M and F	WB	450 K	Differential methylation identified in *TXNIP* and *DQX1* genes in T2D subjects compared to controls.
Kulkarni et al. (85)	Mexican-American	T2D = 174 Controls = 676	M and F	PB	450 K	*TXNIP, ABCG1, SAMD12* associated with T2D, FBG, and HOMA-IR.
Soriano-Tarraga et al. (86)	Caucasian, (Spain)	^a^T2D = 151 Controls = 204 ^b^T2D = 59 (BISMAR cohort) Controls = 108 ^b^T2D = 63 (REGICOR cohort) Controls = 582	M and F	WB	450 K	One differentially methylated region in the *TXNIP* gene, replicated in 2 independent cohorts.
Florath et al. (87)	German	^a^T2D = 154 Controls = 835 ^b^T2D = 87 Controls = 527	M and F	WB	450 K	Differential methylation of *TXNIP* associated with T2D in discovery and replication cohorts.
Jeon et al. (88)	Korean	^a^High-glucose group- 8 T2D = 5 Controls = 13 ^b^T2D = 220 Controls = 220	M and F	PB	^a^450 K ^b^Bisulfite Pyrosequencing	^a^MSI2 hypomethylated by 11% in T2D cases and 7% in high glucose cases (*p*-value = 0.038). CXXC4 hypomethylated by 15% in T2D cases (*p*-value = 0.044), and 12.8% in high glucose cases (*p*-value = 0.033).^b^MSI2 hypomethylation significantly correlated with T2D.
Yuan et al. (89)	European	^a^T2D = 23 Controls = 31 ^b^T2D = 42 Controls = 221	M and F	WB	^a^MeDIP-seq and ^b^450 K	Two DMS within a 2 kb region upstream of the transcriptional start site of the *MALT1* gene on T2D subjects compared to controls.
Matsha et al. (90)	South African, mixed ancestry.	T2D = 3 Prediabetes = 3 Controls = 3	F	PB	MeDIP-seq	1,415 DMS in the promoter regions of T2D subjects compared to normoglycaemic controls. Genes associated with cell surface signaling, glucose transport, insulin signaling, pancreas development, and the immune system.
Pheiffer et al. (11)	South African, mixed ancestry.	T2D = 3 Prediabetes = 3 Controls = 3	F	PB	MeDIP-seq	3,081 DMS in T2D and prediabetic subjects occurred within non-promoter regions, including sites encoding miRNAs.

a Discovery cohort;

b *Validation cohort; DMS, differentially methylated sites; F, female; FBG, fasting blood glucose; FI, fasting insulin; HbA1c, glycated hemoglobin A1c; HOMA-IR, homeostatic model assessment-insulin resistance; M, male; MeDIP-seq, Methylated DNA immunoprecipitation sequencing; PB, Peripheral blood; T2D, Type 2 Diabetes; WB, Whole blood; 450 K, Infinium Human-Methylation450 BeadChip*.

### Microarray-based Methylation Assays

Microarray-based methylation assays use the ratio between hybridization intensities of DNA samples before and after digestion with a cocktail of methyl-sensitive restriction enzymes to generate quantitative methylation scores (91). This technique was used in one of the first GWAS to compare T2D-associated genome-wide methylation alterations in human blood. Toperoff et al. assessed pooled, peripheral blood DNA methylation in a Jewish cohort of 710 T2D and 459 control subjects (79). Their analysis covered 1 461 753 DNA genomic fragments containing 3,359,645 CpG methylation sites and results showed that differentially methylated sites were enriched in genomic regions that had previously been associated with T2D. The most significant methylation differences between T2D and control subjects mapped to the *SLC30A8, TCF7L2, FTO, potassium voltage-gated channel subfamily KQT member 1* (*KCNQ1*), *thyroid adenoma associated protein* (*THADA*), and jux*taposed with another zinc finger protein 1* (*JAZF1*) genes (79). The authors validated these methylation changes using bisulfite sequencing, which also revealed that hypomethylation of a CpG site in the first intron of the *FTO* gene, was significantly associated with T2D risk. Furthermore, these findings were reproduced by the same group in an independent population cohort (Jerusalem LRC longitudinal Study) of young individuals who later developed T2D, indicating that hypomethylation of specific genomic sites may be an early risk factor that predisposes individuals to T2D later in life.

### Beadchip Arrays

Bead array-based DNA methylation analysis is designed to provide single-base resolution and quantitative evaluation of specific cytosines in multiple samples (92). The Infinium HumanMethylation BeadChip was developed by Illumina and interrogates over 485,000 methylation sites and covers 96% of CpG islands, as well as additional island shores (i e., regions flanking 2 kb of CpG islands) (93). Using the HumanMethylation450 BeadChip, Chambers et al. investigated T2D-associated DNA methylation alterations in peripheral blood from 2,664 Indian Asians and replicated the study findings in 1,141 Europeans (80). Differentially methylated CpG sites were identified within 853 genes in individuals with T2D, including known T2D-associated loci, *TCF7L2, FTO*, and *KCNQ1*. The authors also found that CpG sites in *thioredoxin-interacting protein* (*TXNIP*), *ATP-binding cassette sub-family G member 1* (*ABCG1*), *phosphoethanolamine/phosphocholine phosphatase 1* (*PHOSPHO1*), *suppressor of cytokine signaling 3* (*SOCS3*), and *sterol regulatory element-binding transcription factor 1* (*SREBF1*) were significantly associated with the future development of T2D (80). In addition, the combined methylation scores for these five loci were associated with future T2D incidence independently of the established T2D risk factors—family history of T2D, physical activity, BMI, waist:hip ratio, HbA1c, and glucose and insulin concentrations.

The loci identified by the Chambers study were later evaluated in an independent cohort (the Botnia prospective study) by Dayeh et al. (81), who confirmed an association between *ABCG1* and *PHOSPHO1* methylation in whole blood and future T2D risk but not *SOCS3, SREBF1*, or *TXNIP*. They found that *ABCG1* hypermethylation positively associated with HbA1c and fasting insulin levels. Furthermore, the methylation status of *ABCG1* could be replicated in the blood of diabetic twins compared to their non-diabetic counterparts (81). The association between *ABCG1* hypermethylation and fasting blood glucose and insulin levels has also been reported in three other GWAS studies (82–85). The *ABCG1* gene encodes a protein involved in cholesterol transport (94). Since cholesterol abnormalities is a hallmark of T2D, it is tempting to speculate that hypermethylation of this gene may modulate circulating cholesterol levels, and thus have an impact on initiation and progression of T2D, as well as T2D-associated cardiovascular complications (94).

Dayeh et al. also found that DNA hypermethylation at the *PHOSPHO1* locus positively correlated with HDL and associated with decreased T2D risk (81). The *PHOSPHO1* gene encodes a hydrolase enzyme and is involved in skeletal and vascular mineralization (95, 96). Cardiovascular calcification is a common consequence of aging, diabetes and hypercholesterolemia, and PHOSPHO1 has thus been marked as an attractive target for cardiovascular therapy (96). The observation that *PHOSPHO1* hypermethylation correlated with HDL and reduced T2D risk provides additional evidence for its candidacy as a diagnostic marker for T2D–associated CVD complications.

Muftah et al. investigated DNA methylation patterns in the whole blood of 123 subjects from an Arab cohort and replicated eight known CpG associations with T2D/BMI identified in Caucasians, including an association of *TXNIP* hypomethylation with T2D (reported by the Chambers study) (19, 80). Hypomethylation of *TXNIP* in T2D subjects has since been reported in four additional studies using HumanMethylation450 BeadChip arrays (84–87). Interestingly, *TXNIP* expression is induced by glucose, as a result of a carbohydrate response element in its promoter and *TXNIP* overexpression has been reported in both diabetic animals and humans (97). Furthermore, *TXNIP* has been linked to vascular complications through its ability to modulate angiogenesis by repressing vascular endothelial growth factor (VEGF) (97). Muftah et al. also identified a significant association between methylation at a novel CpG site within the *DEAQ-box RNA dependent ATPase* 1 (*DQX1*) gene and T2D in both the Arab and Caucasian cohorts (19). *DQX1* encodes an RNA-dependent ATPase, which is highly expressed in the liver and muscle, however its role in T2D remains to be elucidated (98).

A family-based study by Kulkarni et al. analyzed the association of DNA methylation at 446,356 sites in peripheral blood from 850 pedigreed Mexican-American individuals (85). They found differential methylation of 51 CpG sites that significantly associated with T2D, 19 with increased fasting blood glucose concentrations and 24 with HOMA-IR (85). Interestingly, the five CpG sites that were most significantly associated with T2D-related traits mapped to three genes, including the previously identified *TXNIP* and *ABCG1* genes, and *Sterile Alpha Motif Domain Containing 12* (*SAMD12*). *SAMD12* has been identified as a target of gene fusion in breast cancer (99), however its role in T2D still needs to be explored.

More recently, Jeon et al. (88) investigated genome-wide DNA methylation changes in peripheral blood related to hypoglycaemia in a longitudinal Korean population-based cohort (88). They identified hypomethylation of two genes, *Musashi RNA-Binding Protein 2* (*MSI2*) and *CXXC-Type Zinc Finger Protein 4* (*CXXC4*), in individuals with T2D and impaired glucose tolerance, compared to healthy controls. They further assessed these findings in an additional cross-sectional replication cohort of subjects with T2D and healthy controls, using targeted pyrosequencing. Here, only *MSI2* hypomethylation could be validated, which significantly associated with T2D. Interestingly, the same association was observed in pancreatic islet DNA from subjects with T2D, indicating that *MSI2* methylation may be biologically relevant. This is in line with expression studies performed by Szabat et al. (100), who demonstrated that *MSI2* could be upregulated in response to lipotoxicity and endoplasmic reticulum (ER) stress, and that knockdown/overexpression of MSI2 in mouse pancreatic beta cells resulted in significantly altered insulin expression, suggesting a potential modulatory role for MSI2 in T2D.

### Methylated DNA Immunoprecipitation Sequencing

Methylated DNA immunoprecipitation sequencing (MeDIP-seq) is a versatile, unbiased approach for detecting methylated DNA and involves the use of a monoclonal antibody that specifically recognizes 5 mC to enrich for methylated DNA, after which the immunoprecipitated fraction can be analyzed by large-scale sequencing (101). This approach is particularly useful because it bypasses the need for bisulfite conversion and is able to distinguish between 5 mC and 5-hydroxymethylcytosine, an oxidation product of 5 mC (101).

Yuan et al. (89) investigated epigenome-wide methylation patterns in whole blood from monozygotic twins discordant for T2D using MeDIP-sequencing, after which the top scoring results were replicated in a separate cohort of twins using the Illumina Human Methylation 450 K array. In the first cohort of twins, they identified T2D-associated differentially methylated regions located within 3,597 genes, which were hypermethylated in two-thirds of cases (89). Furthermore, 30% of the differentially methylated regions could be replicated in the additional twin cohort. Importantly, the top two differentially hypermethylated regions identified in the study were found to reside within a 2 kb region upstream of the transcriptional start site of the *mucosa-associated lymphoid tissue lymphoma translocation protein 1* (*MALT1*) gene. Studies in *MALT1* knockout mice have demonstrated its critical roles in antigen- receptor-induced activation of NF-κB (89). NF-κB has well established roles in T2D–associated chronic inflammation, however, the effects of *MALT1* hypermethylation or transcript depletion on NF-κB signaling and associated inflammation in humans has not yet been explored (102). In the same study, Yuan et al. also identified hypermethylation in the promoter region of the *G-protein receptor 6* (*GPR6*) gene, encoding a member of the G protein-coupled receptor family of transmembrane receptors. Interestingly, *GPR6* knockout mice exhibit hyperphagia-induced obesity and higher liver triglyceride content, plasma insulin, and leptin levels compared to wild-type mice (103). These findings suggest that GPR6 plays a role in the regulation of food intake and body weight, and may thus be an important molecular target for obesity or hyperphagia.

Matsha et al. (90) performed GWAS analysis on DNA methylation patterns in peripheral blood from a small cohort of South African women of mixed ethnic ancestry, consisting of 3 subjects with T2D, 3 with pre-diabetes, and 3 with normoglycaemia. They identified 1,415 differentially methylated sites in the promoter regions of T2D subjects compared to normoglycaemic controls, of which over 80% were hypermethylated, including the following genes: *B-Cell CLL/Lymphoma 3* (*BCL3*), *Interleukin 23 Subunit Alpha* (*IL23A*), *F2R Like Trypsin Receptor 1* (*F2RL1*), *S100 Calcium Binding Protein A12* (*S100A12*), *TNF Receptor Superfamily Member 10b* (*TNFRSF10B*), *NIMA Related Kinase 6* (*NEK6*), *Ring Finger Protein 31* (*RNF31*), *Solute Carrier Family 35 Member B2* (*SLC35B2*), and *Interleukin 1 Receptor Associated Kinase 1 Binding Protein 1* (*IRAK1BP1*). Interestingly, when grouped according to chromosomal location it was found that, compared to controls and pre-diabetic subjects, individuals with T2D had hypermethylated regions that were more common in chromosomes 3, 6, 11, 13, and 17, while more hypomethylated methylated regions were found in chromosome 1. Furthermore, these identified hypermethylated regions mapped to pathways related to T2D, including cell surface signaling, glucose transport, insulin signaling, pancreas development, and the immune system, whereas hypomethylated regions related to the pro-inflammatory NF-κB cascade, as well as metabolism pathways for polyunsaturated omega-6 fatty acids, linoleic acid, and arachidonic acid (90). Interestingly, excess consumption of polyunsaturated fatty acids, particularly found at high concentrations in the western diet, can result in increased inflammation and contribute to the onset of chronic diseases including obesity and T2D (104). Importantly, Matsha et al. (90) demonstrated that linoleic acid and arachidonic acid metabolism pathways were also associated with hypomethylated differentially methylated regions in subjects with prediabetes compared to controls, suggesting that alterations in the methylation state of these genes may occur before the onset of overt T2D.

An additional investigation by the same group focused on identifying T2D-associated DNA methylation changes in intergenic regions compared to promoter and gene body regions, as it now appreciated that methylation within intergenic regions regulate RNA processing, as well as high-copy interspersed or tandem DNA repeats (10). Using peripheral blood DNA from the same South African patient cohort as described above (90), they showed increased DNA methylation in intergenic regions compared to gene body and promoter regions (11). Furthermore, 3,081 of the differentially methylated regions were associated with microRNAs. Importantly, a subset of miRNAs identified in the study, including miR-9, miR-34, miR-124, and miR-1297, have already been linked to T2D and associated traits in human and animal diabetic models. Since dysregulated miRNAs have an established role in T2D (105), those identified by Pheiffer et al. merit further evaluation as novel disease risk biomarkers.

## Interactions Between Genetics and Epigenetics in T2D

There is evidence to suggest that single nucleotide polymorphisms (SNPs) may be associated with altered epigenetic signatures (106). Indeed, it has been suggested that up to 25% of all SNPs in the genome either introduce or remove CpG sites (106, 107). In this regard, CpG-SNPs have been suggested to be a potential mechanism through which SNPs affect gene function via epigenetics, highlighting the complex interaction between genetics and epigenetics (107). While CpG-SNPs have been reported in numerous obesity-associated genes, few studies have examined the association between SNPs and T2D risk through effects on DNA methylation (108).

An investigation of T2D-associated DNA methylation candidates reported in this review revealed that 20 genes were indeed associated with SNPs. *FTO*, and *TCF7L2* have been deemed two of the most important T2D susceptibility genes to date (54–57, 109, 110), while additional SNPs in *MCP-1, SLC30A8, GCK, PRKCZ, GIPR, IGFBP-7, PTPN1, PPAR*γ, *KCNQ1, BCL11A, CALM2, CRY2, CAMK1D, THADA, ABCG1, SOCS3, SREBF1, TXNIP* have either been associated with glycaemic traits, T2D or risk of T2D-linked complications (63, 64, 110–119). It is still unknown whether the above associated SNPs may directly cause differential DNA methylation of genes that contribute to the pathogenesis of T2D, or if SNPs within regulatory regions change the affinity and/or binding of transcription factors, which in turn influence the recruitment of epigenetic machinery. Future work should be directed at combining genetic information and methylation marks when comparing individuals with T2D to those without disease.

Interestingly, SNPs may also be associated with altered global DNA methylation. Matsha et al. performed genetic screening of polymorphisms in the *nitric oxide synthase 3* (*NOS3*) gene using peripheral blood from South African subjects with T2D, pre-diabetes or normoglycaemia, and reported that the *NOS3* G894T polymorphism was independently associated with global DNA methylation. NOS3 has previously been reported to be affected by supplementation with folate, a dietary methyl donor (120). Although the potential role played by NOS3 in global DNA methylation is unclear, it encodes an enzyme involved in endothelial function and may thus potentially contribute to T2D-associated vascular complications (121).

## Challenges and Limitations in DNA Methylation Studies

The current review highlighted variation in the outcome of DNA methylation studies, however, it is important to note that the range of methods employed to measure DNA methylation is vast, as are the sources of DNA (cell type), DNA isolation method and methods of data analysis (122). This large heterogeneity complicates the direct comparison of findings between studies, particularly for those published more than a decade ago, as the epigenetics field is expanding at such a rapid rate. The standardization of experimental and analysis approaches, as well as internal and external validation of study findings will be an important step in improving the reproducibility and biological relevance of these findings.

The inability of some of the reported studies to replicate methylation associations could be explained by differences in the groups of participants analyzed, as the majority of findings emanated from small cross-sectional or case-control studies in varying populations. To advance reproducibility in different populations, more robust longitudinal studies are required, which would involve prospective recruitment of a large cohort of healthy individuals at baseline, and the follow-up of these individuals over several decades to track T2D incidence. However, due to higher costs and study duration, longitudinal studies for complex diseases such as T2D still remain scarce. The only longitudinal studies reported in this review were that of Chambers et al. (80) and Jeon et al. (88). Interestingly, differentially methylated genes identified in the Chambers study were replicated in cross-sectional studies (*TXNIP, ABCG1, PHOSPHO1*, and *SREBF1*) (19, 81–87). The ability of these changes to be captured across studies of different time lengths may be attributed to the stable nature of DNA methylation marks after disease onset.

An additional limitation in some of the reported studies was failure to include in-depth demographic, lifestyle, and health data and consequently, lack of consideration or adjustment for potential confounding factors. This is crucial for data interpretation as it is now widely appreciated that DNA methylation signatures vary with gender, age, and ethnicity and are sensitive to many environmental influences (32). Indeed, in cases where gender was considered, differences in DNA methylation patterns were reported for *BCL11A, GCK, IGFBP-7*, and *PTPN1* (44, 46, 49). These observations are consistent with findings on gender-specific DNA methylation marks in other diseases, such as *PLA2G7* in cardiovascular disease and *MTHFR* in schizophrenia (123, 124). Furthermore, gender-specific differences in glucose homeostasis and T2D risk have been reported, which may be related to the levels of sex-hormones such as estrogen and testosterone (125). The confounding effect of chronic medication was also highlighted by Matsha et al. (30), who reported an association between global DNA hypomethylation in T2D individuals and the use of glucose-controlling agents. Indeed, the widely used anti-diabetic drug, metformin, was recently shown to promote global DNA methylation in cancer cell lines by modulating the intracellular ratio of S-adenosylhomocysteine (SAH) and S-adenosylmethionine (SAM) (126). Thus, the observed methylation differences and associations between methylation changes and T2D risk might be confounded by medications, such as metformin, which should be taken into consideration in future studies. Furthermore, these findings may offer opportunities for the use of DNA methylation for monitoring of management and response to T2D medications.

DNA methylation varies with cell type and thus cellular homogeneity within a tissue is an important characteristic for a DNA methylation biomarker. While the finding in this review strengthen the candidacy of blood-based markers, it is important to note that blood exhibits cellular heterogeneity, as it consists of a wide variety of cell types including erythrocytes, basophils, neutrophils, eosinophils, monocytes, lymphocytes, natural killer cells, and platelets (127). Each of these cells possess a unique epigenetic signature and this can lead to variation between studies. Only one study reported in this review controlled for the estimated proportion of different blood cell types, and indeed proved that epigenetic heterogeneity in whole blood cell constituents impacts on data interpretation (31). Thus, it is plausible that differences in cell composition between groups may drive false associations or mask potential differences between groups. In this regard, there are several additional methods that can be used to avoid potential confounding effects of the blood cell composition, such as the measurement of DNA methylation in individual cell types following sorting of the cells, adjustment for direct measured cell count or the use of *post-hoc* regression models, as described by Houseman et al. (128).

It is important to note that while DNA methylation in gene promoters has consistently been linked with gene silencing, some studies could not correlate promoter methylation with gene or protein expression (49). This could either be a result of DNA methylation at the reported CpGs being ineffective to reduce transcript levels, or due to alternative transcriptional or post-transcriptional influences. Indeed, a growing body of evidence suggests that miRNAs and histone modifications are also highly involved in T2D pathogenesis, for which comprehensive reviews have been published (105, 129, 130).

## Conclusions and Future Perspectives

**Figure 1:**
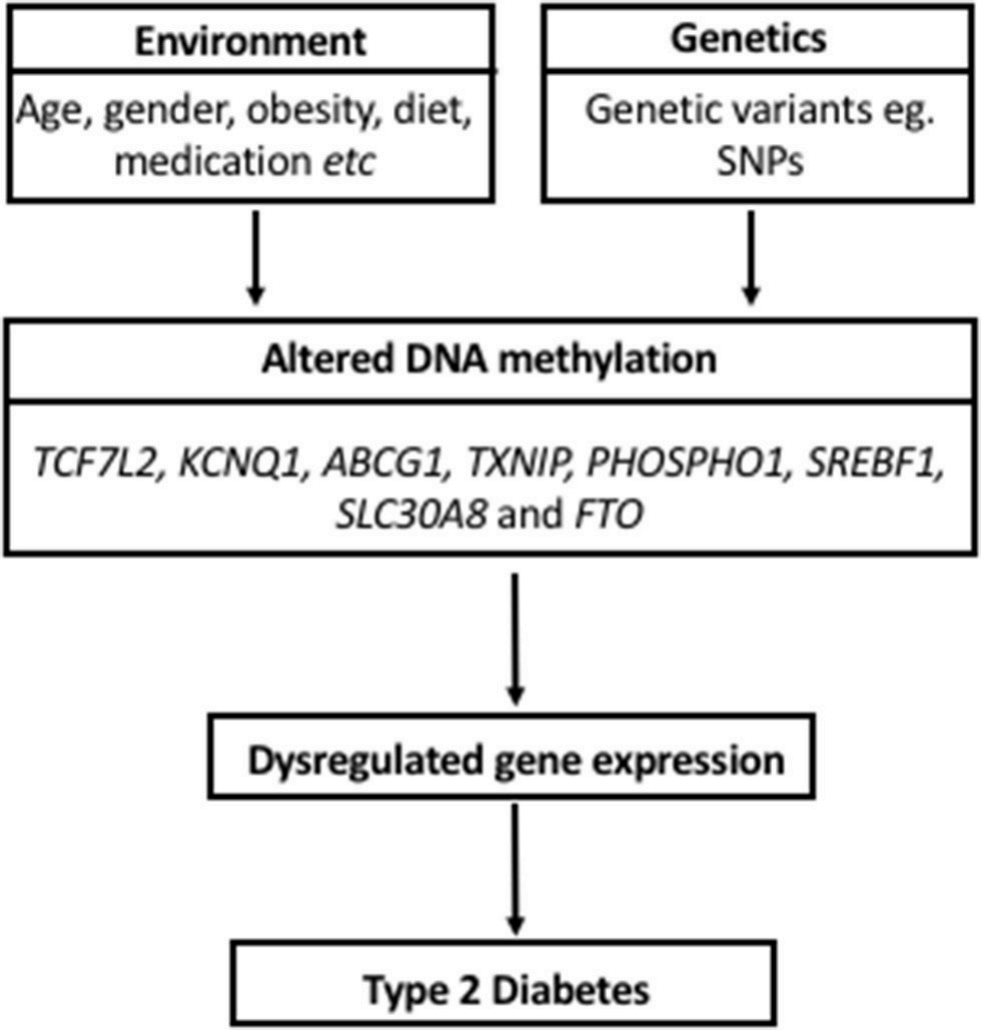
Model proposing a role for DNA methylation in the pathogenesis of Type 2 Diabetes and its interaction with environmental factors and genetics.

The current review identified 37 articles investigating DNA methylation markers for T2D detection or risk evaluation, using DNA isolated from blood. Based on reproducible findings from the reviewed studies in different population groups, differentially methylated sites in *TCF7L2, KCNQ1, ABCG1, TXNIP, PHOSPHO1, SREBF1, SLC30A8*, and *FTO* are potentially associated with T2D and their predictive powers may hold irrespective of different genetic backgrounds and different lifestyle or environmental pressures. A model for the role of these DNA methylation alterations in the pathogenesis of T2D is depicted in Figure 1. Although these alterations were detected in blood, which is not an insulin-responsive tissue, the implicated genes have been shown to play a role in critical biological processes that are deregulated during T2D development, including energy intake and expenditure (*FTO*), lipogenesis and glycolysis (*SREBF1*), glucose homeostasis and carbohydrate metabolism (*TXNIP, TCF7L2*), lipid transport (*ABCG1*) pancreatic insulin secretion (*SLC30A8, KCNQ1*), and cardiovascular function (*PHOSPHO1*). It is thus plausible that these blood-based epigenetic markers mirror tissues with deteriorated metabolic function, and are prime non-invasive candidates for T2D biomarkers.

Some limitations of the current review are the exclusion of studies that were not published in English and the use of only four databases of published literature. Positive publication bias also should be considered, as studies with negative findings may not have been published. Finally, the current review only focused on DNA methylation, given the large scope of studies already published in this field. Although it was beyond the scope of this review, we cannot rule out other possible epigenetic biomarker candidates of T2D, such as non-coding RNAs and histone modifications, for which evidence is rapidly accumulating (105, 129, 130).

The major strength of this review is the central focus on blood-based DNA methylation signatures in T2D, which will have important implications for the development of non-invasive T2D screening tests, given the difficulty in accessing T2D-associated tissues, particularly for longitudinal studies. To the best of our knowledge, this is the only review solely examining associations of T2D with DNA methylation profiles in peripheral blood. We also highlighted specific methylation patterns that associated with T2D risk factors such as BMI, HbA1c levels, and HDL/LDL, which further supports the hypothesis that profiling DNA methylation in blood could be used to monitor high risk individuals and delay or prevent T2D by facilitating early intervention strategies. In this regard, the candidate markers in this review need to be further validated in additional prospective study cohorts and tested in large screening populations by high quality studies. At present, no epigenetic biomarkers for T2D have yet entered clinical trials, however, there is hope that initiatives such as next generation sequencing and the use of longitudinal study designs, will uncover important predictive T2D biomarkers.

## Author Contributions

TW designed the study and extracted the data. TW wrote the manuscript. CP and RJ corrected the manuscript. All authors read and approved the final manuscript.

### Conflict of Interest Statement

The authors declare that the research was conducted in the absence of any commercial or financial relationships that could be construed as a potential conflict of interest.

## Abbreviations

T2D: Type 2 diabetes
FFAs: free fatty acids
5mC: 5-methylcytosine
CpG: Cytosine-guanine dinucleotides
GWAS: genome-wide association studies
ELISA: enzyme-linked immunosorbent assay
HDL: high-density lipoprotein
NOS3: nitric oxide synthase 3
LINE-1: long interspersed nuclear element-1
LDL: low-density lipoprotein
Alu: Arthrobacter luteus
MeDIP: methylated DNA immunoprecipitation
FTO: obesity-associated protein
PPARγ: peroxisome proliferator–activated receptor gamma
PDK4: pyruvate dehydrogenase lipoamide kinase isozyme 4
GCK: glucokinase
PRCKZ: protein kinase C zeta
GIPR: gastric inhibitory polypeptide receptor
IGFNP-7: insulin-like growth factor-binding protein 7
PTPN1: protein tyrosine phosphatase, non-receptor type 1
MCP-1: monocyte chemoattractant protein-1
SLC30A8: solute carrier family 30 member 8
BCL11A: B-cell lymphoma/leukemia 11A
TCF7L2: transcription factor 7-like 2
CALM2: calmodulin 2
CRY2: CRY2 cryptochrome circadian regulator 2
CMAK1D: Ca2+/calmodulin-dependent protein kinase 1 subfamily of serine/threonine kinases
TLR2: toll-like receptor 2
TLR3: toll-like receptor 3
FFAR3: free fatty acid receptor 3
PI3K: phosphatidylinositol 3-kinase
GLUT4: glucose transporter type 4
SNP: single nucleotide polymorphisms
HOMA-IR: homeostatic model assessment for insulin resistance
ROS: reactive oxygen species
GI: gastrointestinal
BMI: body mass index
SCFA: short chain fatty acids
FFARs: free fatty acid receptors
OGTTs: oral glucose tolerance tests
KCNQ1: potassium voltage-gated channel subfamily KQT member 1
THADA: thyroid adenoma associated protein
JAZF1: juxtaposed with another zinc finger protein 1
TXNIP: thioredoxin-interacting protein
ABCG1: ATP-binding cassette sub-family G member 1
PHOSPHO1: phosphoethanolamine/phosphocholine phosphatase 1
SOCS3: suppressor of cytokine signaling 3
SREBF1: sterol regulatory element-binding transcription factor 1
HbA1c: hemoglobin A1c
VEGF: vascular endothelial growth factor
DQX1: DEAQ-box RNA dependent ATPase 1
SAMD12: Sterile Alpha Motif Domain Containing 12
MALT1: mucosa-associated lymphoid tissue lymphoma translocation protein 1
GPR6: G-protein receptor 6
TG: triglyceride
BCL3: B-Cell CLL/Lymphoma 3
IL23A: Interleukin 23 Subunit Alpha
F2RL1: F2R Like Trypsin Receptor 1
S100A12: S100 Calcium Binding Protein A12
TNFRSF10B: TNF Receptor Superfamily Member 10b
NEK6: NIMA Related Kinase 6
RNF31: Ring Finger Protein 31
SLC35B2: Solute Carrier Family 35 Member B2
IRAK1BP1: Interleukin 1 Receptor Associated Kinase 1 Binding Protein 1.
