# Corrigendum: Blood-Based DNA Methylation Biomarkers for Type 2 Diabetes: Potential for Clinical Applications

**DOI:** 10.3389/fendo.2019.00001

**Published:** 2019-01-22

**Authors:** Tarryn Willmer, Rabia Johnson, Johan Louw, Carmen Pheiffer

**Affiliations:** ^1^Biomedical Research and Innovation Platform, South African Medical Research Council, Tygerberg, South Africa; ^2^Division of Medical Physiology, Faculty of Health Sciences, Stellenbosch University, Tygerberg, South Africa; ^3^Department of Biochemistry and Microbiology, University of Zululand, Kwa-Dlangezwa, South Africa

**Keywords:** global DNA methylation, gene-specific DNA methylation, genome-wide DNA methylation, blood, type 2 diabetes, biomarkers

In the original article, there was an error. “Colombian” was misspelled as “Columbian.”

A correction has been made to the **Introduction**, **Global DNA Methylation Studies**, paragraph two.

“Luttmer et al. quantified global DNA methylation levels in peripheral blood leukocytes of 738 individuals from the Netherlands Hoorn Study cohort and reported a progressive decrease in global DNA methylation in individuals with T2D compared to those with impaired glucose tolerance and normoglycaemia. Moreover, DNA hypomethylation in these subjects was independently associated with hyperglycaemia and high-density lipoprotein (HDL) cholesterol (28). In contrast, a Colombian study using a smaller patient group, observed a global increase in DNA methylation in 44 subjects with T2D compared to 35 healthy controls, which correlated with the percentage of glycated hemoglobin A1c (HbA1c) (29). Similar findings were reported by Matsha et al. using a South African population consisting of 158 individuals with T2D, 119 with dysglycaemia, and 287 healthy controls. They showed that levels of global DNA methylation were higher in individuals with impaired glucose tolerance or treatment-naïve T2D compared to those with normoglycaemia (29, 30). Interestingly, no difference in global DNA methylation was observed between diabetic individuals on treatment and normoglycaemic subjects, prompting the authors to speculate that glucose management caused the reversal of aberrant DNA methylation patterns during T2D (30).”

The authors apologize for this error and state that this does not change the scientific conclusions of the article in any way. The original article has been updated.

